# Penetrating injury with an arrow impacted in the neck in rural Tanzania, a case report

**DOI:** 10.1016/j.ijscr.2022.107133

**Published:** 2022-04-30

**Authors:** Allyzain Ismail, Neelam Ismail, Athar Ali, Rashid Mayoka, Winfrid Gingo, Fassil Gebreegziabher

**Affiliations:** a The Aga Khan University, East Africa Medical College, Tanzania; b The Aga Khan Hospital, Dar-es-Salaam, Tanzania; c Saint Francis Referral Hospital, Ifakara, Tanzania

**Keywords:** Penetrating neck injury, Arrow impacted, Surgical exploration, Case report

## Abstract

**Introduction and importance:**

Penetrating neck injuries refer to neck injuries that penetrate through the platysma layer which can cause life-threatening injuries to the aerodigestive and neurovascular systems. Currently penetrating neck injuries are mainly due to modern firearms however penetrating neck injuries due to arrows are still seen, although rare, in rural areas among developing countries. Management depends on hemodynamic stability, signs of structural damage and zone of neck involved.

**Case presentation:**

19-year-old male presenting with a penetrating arrow impacted in his neck following an attack by cattle raiders who was otherwise stable of presentation. Underwent preoperative imaging with removal of the arrow under general anaesthesia via surgical exploration.

**Clinical discussion:**

We concur with the recommendation to first obtain adequate imaging to establish degree of injury as well as for operative planning. Removal of impacted arrow should not be carried out blindly but rather in a controlled environment under general anaesthesia via surgical exploration.

**Conclusion:**

Penetrative arrow injuries to the neck are an archaic problem that can be dealt with via modern surgical principles towards penetrating neck injuries. Unstable patients warrant emergent exploration however stable patients can first be worked up appropriately to ascertain degree of injury. Impacted arrows can then be removed safely under general anaesthesia rather than risk further injury to critical structures via blind removal.

## Highlights

• Penetrating neck injury by an impacted arrow in rural countryside
• Application of surgical principles in penetrating neck injury due to an arrow
• Assessing relevant vascular anatomy via ultrasound due to financial constraints
• Successful surgical exploration and safe removal without postoperative complications

## Abbreviations

ATLS: Advanced Trauma Life Support
CT: Computed Tomography
PNI: Penetrating neck injuries

## 1 Introduction and importance

Penetrating neck injuries (PNI) refer to neck injuries resulting from gunshot wounds, stab wounds, or penetrating debris that penetrate through the platysma layer which can cause life-threatening injuries to the aerodigestive and neurovascular systems. In the current day and age PNI are mainly due to modern firearms however injuries due to arrows are still seen, although rare, in rural areas among developing countries. It has been seen among individuals due to clash in clans, conflicts between farmers and herders, and unlawful cattle raiding, however believed to be underreported due to majority of the cases occurring in rural areas [1]. Severity of injury depends on the distance from which the arrow was shot, degree of penetration, involvement of vital structures, and the use of poison to coat the tip of the arrow head [2].

The neck region is highly susceptible to threatening injuries due to the presence of vital structures connecting the thorax to the head with minimal protection from a bony covering other than the cervical vertebra shielding the spinal cord structures. Mortality associated with PNI is at about 6% to 10% with majority due to active massive bleeding or an expanding hematoma causing compression effect [3], [4]. Management involves initial attempts at stabilisation via utilization of Advanced Trauma Life Support (ATLS) guidelines. This is followed by decision for immediate surgical exploration verses adequate prior radiological work up to decide on surgical intervention or conservative observation depending on hemodynamic stability, signs of structural damage and zone of neck involved [5]. The neck is divided into 3 zones with zone I from sternum to cricoid cartilage, zone II from cricoid cartilage to base of skull and zone III from base of skull to angle of the mandible [6]. Patients who are hemodynamically and respiratory stable should undergo diagnostic imaging with computed tomography (CT) with angiography for evaluation of the injury the most recommended modality [7].

We report a case of a young cowhand who presented with a penetrated arrow lodged into his neck following altercation with cattle raiders who underwent successful neck exploration and removal of the arrow. We describe our experience from a resource limited setting from rural Ifakara in Tanzania highlighting our approach to an archaic problem with arrows via utilization of modern surgical principles towards PNI. This paper has been reported in line with the SCARE 2020 criteria [8]. This article has been registered with the Research Registry with identification number researchregistry7763 and can be found through the following hyperlink Browse the Registry - Research Registry.

## 2 Case presentation

A 19-year-old male referral from Mahenge District Hospital presented to the emergency department at Saint Francis Referral Hospital with a penetrated arrow lodged in his neck from an attack by cattle raiders 10 h prior with no other injury reported. Reported history of blood loss from injury site which was controlled via compression but otherwise no history of continuous active bleeding, no difficulty speaking, no dysphagia, no change in voice nor difficulty in breathing. He otherwise had no drug allergies, no significant family history of disease, no prior surgeries and did not smoke or drink alcohol. He was initially taken to the nearest primary health centre whereby they carefully cut off the stump of the arrow, dressed the wound and referred to our centre for further management.

**Fig. 1:**
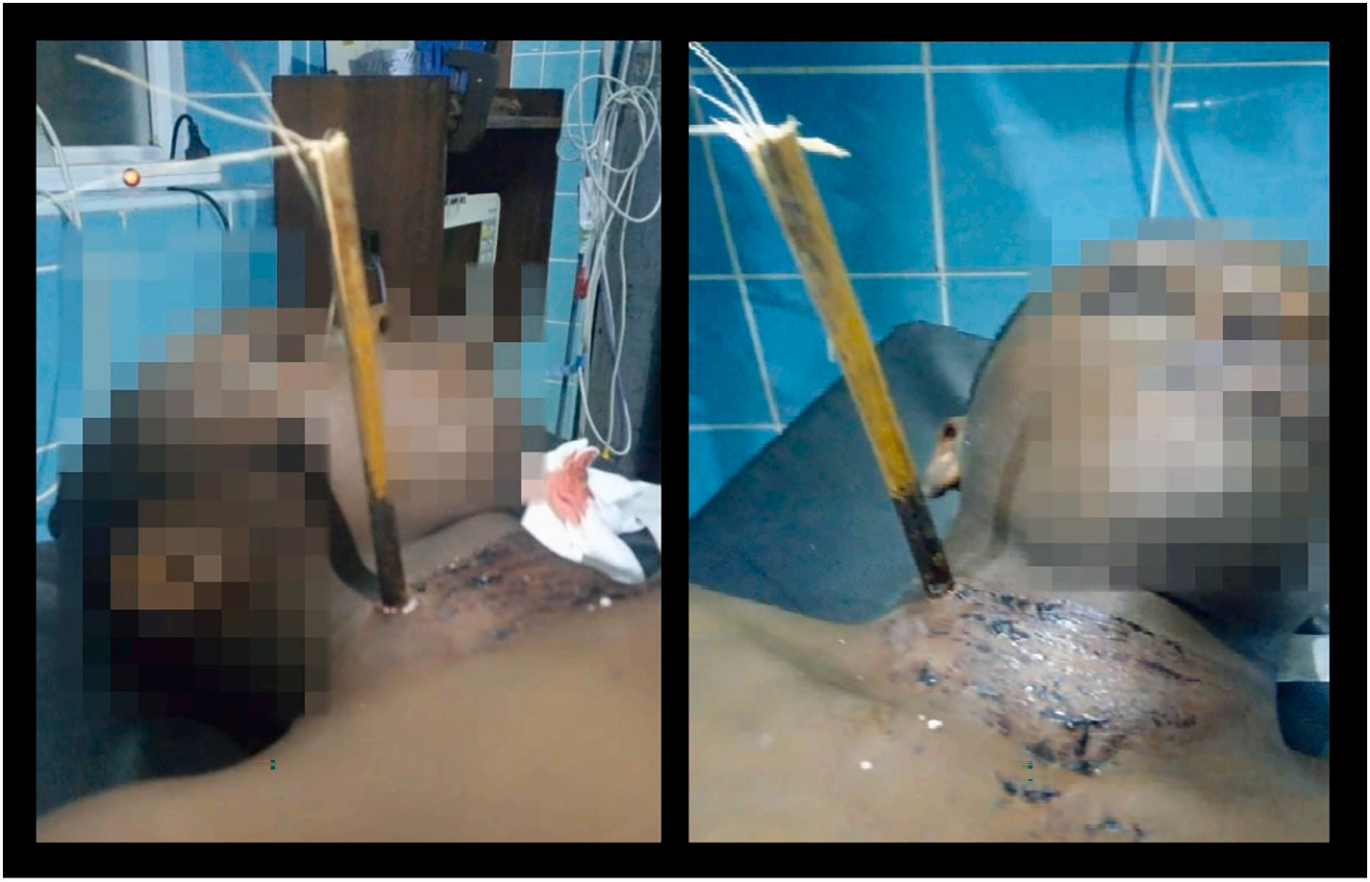
Arrow impacted into the right side of the neck.

On examination he was alert, oriented, not pale, not cyanosed with stable vitals. He had an arrow impacted into the right sided lateral aspect of the anterior triangle of his neck. The entry point was lateral to the cricoid cartilage corresponding to the axial plane of C6 vertebra hence concluded as a zone II PNI (Fig. 1). There was no active bleeding from puncture site, no gross neck swelling, no hoarseness in voice, no bleeding from mouth or nose, no bubbling from injury site, no stridor nor respiratory distress and no features of neurological deficit. On palpation there was tenderness around injury site but no crepitus felt, no emphysema with carotid pulses palpable and intact range of motion and power of the right upper limb. Rest of systemic examinations were normal.

**Fig. 2:**
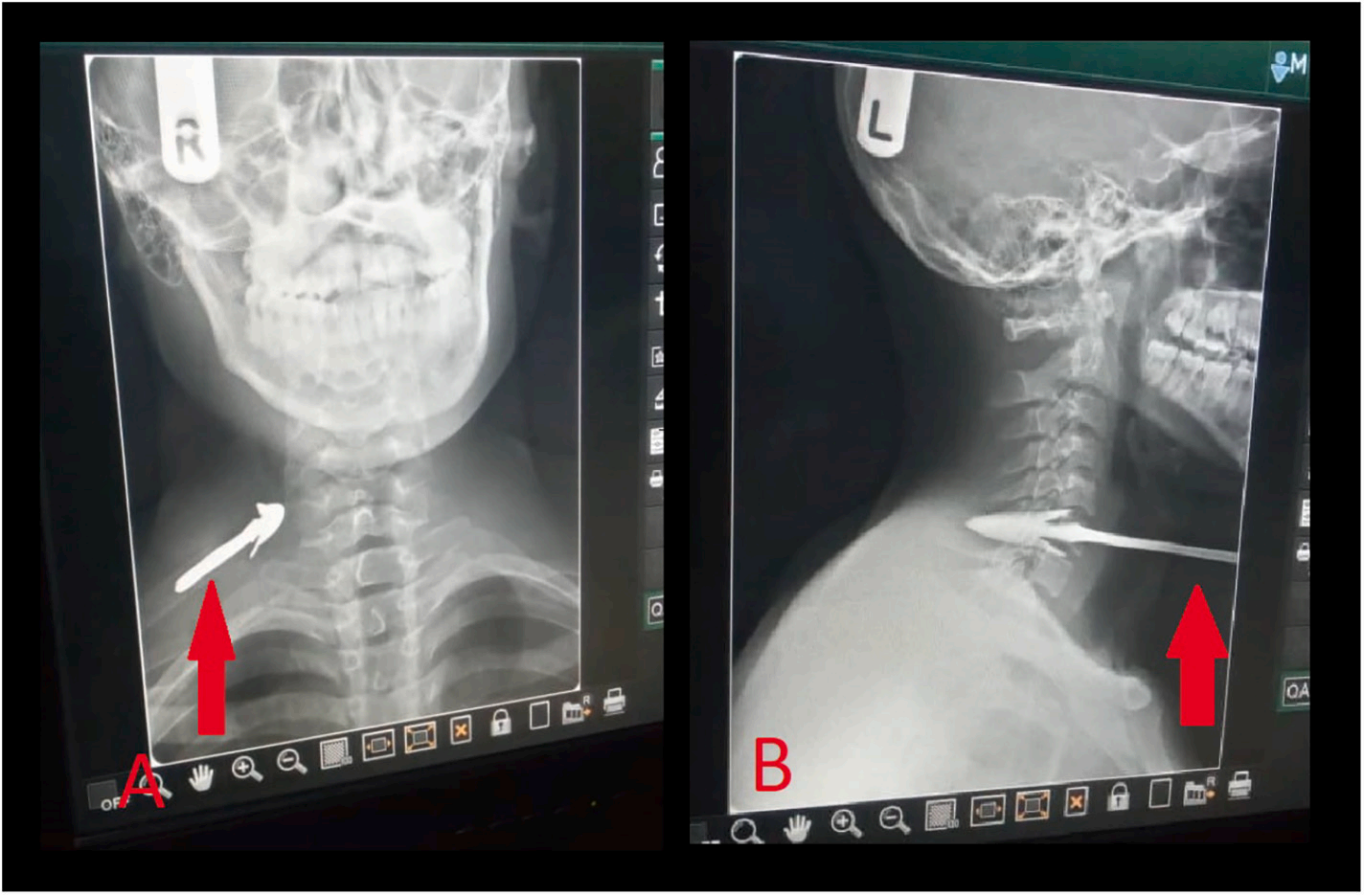
X-ray of the neck. A – Anteroposterior view showing the arrow heading towards lateral aspect of the cervical spine. B – Lateral view showing the arrow head at around C6 level.

**Fig. 3:**
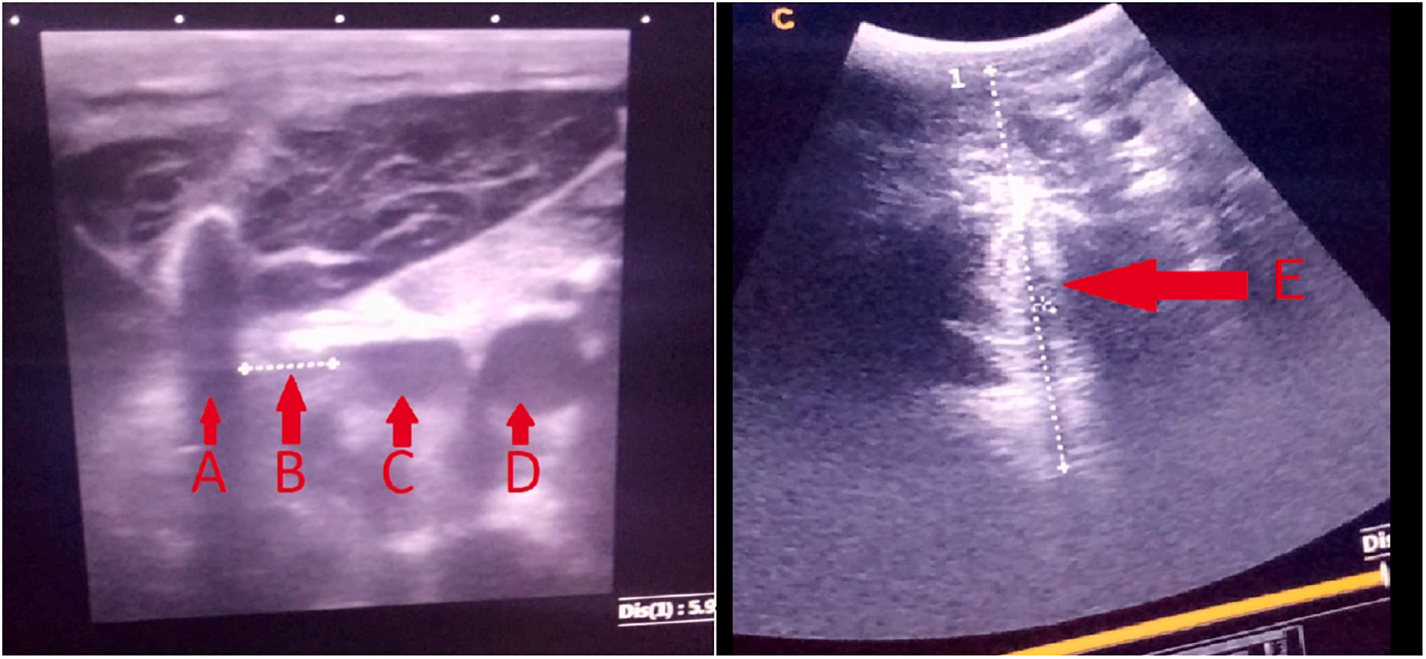
Ultrasound imaging of the neck showing an impacted arrow in close proximity to vitals vascular structures. A – Impacted arrow. B – Distance from edge of arrow to internal jugular vein measuring 5 mm and from arrow to common carotid artery measuring 1 cm. C – Internal jugular vein. D – Common carotid artery. E – Depth of arrow penetration measuring 8 cm deep.

Was planned for a CT angiography of the neck however not carried out due to financial constraints hence an anteroposterior and lateral x-ray (Fig. 2) revealed an arrow lodged in the neck with the arrow head appearing to travel lateral and posterior to the sixth cervical vertebrae. Bedside doppler ultrasound performed by the consultant emergency physician estimated the depth of the penetration to be around 8 cm and the arrow situated 5 mm lateral to the internal jugular vein and 1 cm lateral to the common carotid artery (Fig. 3). He was reviewed by a consultant general surgeon, due to lack of availability of a vascular and head and neck surgeon, and was prepared for emergent neck exploration and arrow removal under general anaesthesia while receiving preoperative antibiotic prophylaxis, analgesia, tetanus prophylaxis and 2 units of standby cross matched blood prepared.

**Fig. 4:**
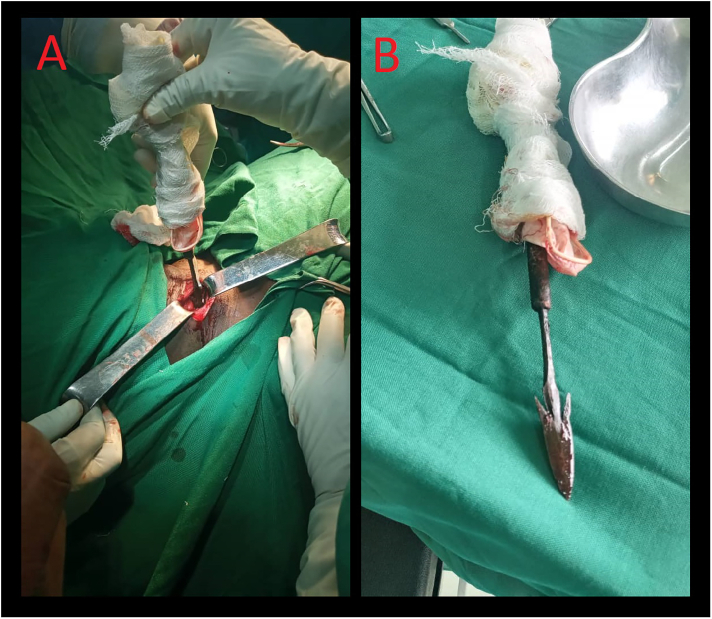
Intraoperative images. A – Incision extended laterally from entry point followed by meticulous dissection to free the arrow tip and arrow head fangs with careful anterior removal of arrow. B – Removed impacted arrow.

Procedure was carried out by 2 consultant general surgeons with a general surgery resident and intraoperatively the incision was extended laterally from the arrow entry point so as to avoid the medial vital vascular structures seen on ultrasound. Dissection was carried out until the arrow tip and fangs of the arrow head were exposed and removed gently (Fig. 4). Findings revealed the arrow to have traversed through the sternocleidomastoid muscle and directing laterally into the posterior triangle with no obvious injury to a neurovascular bundle, the trachea or oesophagus. The sternocleidomastoid muscle was repaired, wound site irrigated with warm saline and closure with sterile dressing placed.

Post operatively was placed on intravenous ceftriaxone 2 g once daily, pain management (intravenous paracetamol 1 g 8 hourly and intramuscular tramadol 100 mg 8hourly), and close monitoring as per postoperative hospital protocols. Day 1 postoperative only complained of mild pain at incision site but could tolerated feeds, speak and talk without difficulty and on examination had stable vitals, a clean wound with no central neurological deficit nor impaired use of the right upper limb. Was observed for another 24 h which was uneventful and was then discharged on oral analgesia (diclofenac 50 mg 8 hourly with pantoprazole 40 mg daily prophylaxis), antibiotics (cefixime 200 mg 12 hourly) and counselled on warning signs. Subsequent follow up visits to the surgical outpatient clinic were uneventful with no complications and has resumed his normal daily life routine.

## 3 Discussion

Arrow injuries to the neck is a rare presentation however application of principles of surgery towards injuries to the neck can be applied. Prehospital management involves immediate transfer to nearest trauma centre, impacted objects to not be removed and cervical spine immobilization only if there are neurological deficits or suspicion for injury in an unexaminable patient [6]. As with our case the impacted arrow was not tampered with and he had no features to suggest cervical injury hence was transported to our facility with only a dressing around the arrow for further management.

At initial evaluation hard and soft signs must be looked for with hard signs reflecting serious injury necessitating immediate surgical exploration. On the other hand, hemodynamic and respiratory stable patients are approached by either the selective zone-based or no-zone approach depending on surgeons' preference and local institutional practicing guidelines [7]. We apply a no-zone approach and as with our case he presented 10 h after injury due to infrastructural logistics in a relatively stable condition involving zone II injury. This did not necessitate immediate exploration however the presence of the impacted arrow regardless of the zone it involved warranted neck exploration after resuscitation and adequate preoperative preparation.

Regardless of approach each recommends the use of CT angiography for stable patients which has high sensitivity and specificity for laryngotracheal, vascular and pharyngoesophageal injuries thus reducing the need for multiple imaging studies to assess each type of injury as well as to assess for important internal structures in close proximity to the injury site [9]. Such information can be used to allow for better decision making regarding the need for observation, further diagnostic evaluation, or surgical exploration. We planned for CT angiography however due to financial reasons had to settle for an X-ray to delineate the impacted portion of the arrow and an ultrasound to assess depth and proximity to vital vascular structures so as to formulate our surgical approach to extracting the arrow safely.

To our knowledge this is the only reported report of a penetrating arrow injury to the neck in East Africa stressing on an earlier point on underreporting of penetrating arrow injuries. However, a few reports from Nigeria have highlighted their individual approaches to penetrating neck arrow injuries with each advocating for removal of the impacted arrow via surgical exploration under general anaesthesia [10]. Another report from Nigeria focusing on penetrating arrow injuries to the head and neck region encouraged removal of impacted arrows via surgical exploration with preoperative CT imaging however also faced similar limitations to do so due to financial constraints hence carried out with preoperative planning based of X-ray imaging only [2]. Through our experience we endorse the notion that an attempt to blindly extract the arrow should be avoided as you may injure vitals structures that are in close proximity. Hence the need for formal surgical dissection under general anaesthesia with meticulous dissection to expose the arrow tip and fangs so as to facilitate safe removal.

## 4 Conclusion

Penetrating arrow injuries are is an archaic problem which is very rarely encountered however the principles towards PNI along with ATLS guidelines on evaluation and resuscitation can be applied to such a case. In our setting we have ongoing conflicts between farmers and cattle herders along with criminal cattle raiding hence believe an underreporting of such cases. We hope through our successful experience to direct practitioners faced with a penetrating neck arrow injury to employ the principles of approach towards PNI as well as attempt removal of the arrow under a controlled environment under general anaesthesia.

## Patient's perspective

I was very concerned when I saw an arrow stuck in my neck and was told to just stay still but felt very anxious all the way until I was operated. However, after the operation other than pain I did not feel anything unusual. I was also shown the arrow that was removed and cannot believe it was in my neck.

## Ethical approval

Case study is exempt from ethical approval in my institution.

## Consent

Written informed consent was obtained from the patient for publication of this case report and accompanying images. A copy of the written consent is available for review by the Editor-in-Chief of this journal on request.

## Guarantor

Dr. Fassil Gebreegziabher

## Sources of funding

This research did not receive any specific grant from funding agencies in the public, commercial, or not-for-profit sectors.

## CRediT authorship contribution statement

A.I: Study conception, production of initial manuscript, collection of data.

N·I: Revision of the manuscript, proofreading.

A.A: Revision of the manuscript, proofreading.

R.M: Production of initial manuscript.

W.G: Revision of the manuscript, proofreading.

F.G: Study conception, revision of the manuscript, proofreading.

Provenance and peer review.

Not commissioned, externally peer-reviewed.

## Declaration of competing interest

None.
